# Extra virgin olive oil inhibits *Helicobacter pylori* growth *in vitro* and the development of mice gastric mucosa lesions *in vivo*

**DOI:** 10.3389/fmicb.2022.961597

**Published:** 2022-08-05

**Authors:** Andrea Celeste Arismendi Sosa, María Laura Mariani, Alba Edith Vega, Alicia Beatriz Penissi

**Affiliations:** ^1^Área de Microbiología e Inmunología, Facultad de Química, Bioquímica y Farmacia, Universidad Nacional de San Luis, San Luis, Argentina; ^2^Instituto de Histología y Embriología “Dr. Mario H. Burgos” (IHEM-CCT Mendoza-CONICET), Facultad de Ciencias Médicas, Universidad Nacional de Cuyo, Mendoza, Argentina

**Keywords:** olive oil, EVOO, hydroxytyrosol, oleuropein, *Helicobacter pylori*, gastric mucosa

## Abstract

*Helicobacter pylori* infection is widespread worldwide, with more than a half of the world population infected. *H. pylori* antibiotic-resistant strains and non-compliance to therapy are the major causes of *H. pylori* eradication failure. The search for new therapies based on plant extracts is a scientific interest field. The present study was conducted to evaluate the effect *in vitro* of extra virgin olive oil (EVOO), hydroxytyrosol (HT), and oleuropein (Olp) against two *H. pylori* strains and the effect *in vivo* of the oral administration of EVOO on the gastric mucosa of BALB/c mice infected with this microorganism. The broth microdilution method assayed the antibacterial *in vitro* activity of EVOO, HT, and Olp against *H. pylori* strains. For *in vivo* studies, male BALB/c mice were infected orally with an *H. pylori* suspension every 72 h. Four groups were used: (1) Control, (2) *H. pylori*-infected (HP), (3) EVOO, and (4) HP + EVOO. Mice were sacrificed at 7, 15, and 30 days. The stomachs were removed and observed under a microscope. Scoring of the degree of erosion was determined. Samples were processed by histological techniques for light microscopy. Macroscopic analysis showed that the presence of small erosions increased, both in number and size, in the infected group. Animals infected and treated with EVOO exhibited the presence of fewer erosions, which decreased in number as the treatment progressed. The mucosa of the control and EVOO groups showed normal histological characteristics at the three times studied. The mucosa of animals infected with *H. pylori* showed disruptions of the lining epithelium, damage to gastric glands, and vasodilation. The mucosa of animals infected with *H. pylori* and treated with EVOO showed morphological characteristics similar to those of normal and EVOO mucosa. For the first time, the current study showed the effect *in vitro* and *in vivo* of EVOO and combined administration of HT and Olp against *H. pylori* using an animal model. Future studies are needed to establish the mechanism of EVOO’s action at the gastric mucosa level to propose this product as a natural antimicrobial agent for the treatment of gastric *H. pylori* infections.

## Introduction

*Helicobacter pylori* is a Gram-negative bacterium that infects about half of the world’s population and about 80% of the population in developing countries (Ranjbar et al., 2017; Alipour, 2021; Siwe et al., 2021). It colonizes the gastric mucosa and triggers pathologic conditions such as peptic ulcer, chronic gastritis, gastric mucosa-associated lymphoid tissue lymphoma (Matongo and Nwodo, 2014; Huang et al., 2017; Park et al., 2018). Besides, in 1994, *H. pylori* was classified as a type I carcinogen by the World Health Organization, since infection with this microorganism is considered the main factor for the development of gastric cancer (Mladenova, 2021). In addition to this, in recent years it has also been associated with various extra-gastric pathologies such as autoimmune, cardiovascular, colonic, respiratory, skin, neurological, and hematological diseases (Ranjbar et al., 2017; Gravina et al., 2020). The infection with this microorganism leads to infiltration of chronic inflammatory cells and accumulation of neutrophil leukocytes in the gastric mucosa, thus leading to continuing inflammation (Palacios-Espinosa et al., 2014). Bacterial eradication therapies involve using bismuth, or a proton pump inhibitor, and two antibiotics, such as amoxicillin, tetracycline, clarithromycin, or metronidazole, in triple or quadruple therapies. However, eradication is not always successful because some patients fail to respond to the treatment or due to antibiotic resistance, where in some regions an increase in resistance to clarithromycin and metronidazole greater than 80% has been reported (Dudley et al., 2017; Malfertheiner et al., 2017; Ozturk et al., 2017; Ranjbar et al., 2017). Because of this, there is a strong demand in the search for new antimicrobial agents. Natural medicine contains active principles such as antimicrobial, anti-inflammatory, antioxidant, and anticancer properties (Baker, 2020). Extra virgin olive oil (EVOO) is obtained by the mechanical pressing of the fruits of the olive tree (*Olea europaea* L.) (Nazzaro et al., 2019) without other treatments, preserving in this way high amounts of phenolic constituents that have beneficial effects on the human health when is part of the diet (Thielmann et al., 2017; Marcelino et al., 2019). EVOO has been associated with reduced incidence of degenerative diseases, such as coronary heart disease and several types of cancer. More specifically, it has been shown that EVOO exerts health benefits mainly, but not only, *via* phenolic constituents such as hydroxytyrosol (HT) and oleuropein (Olp) (Waterman and Lockwood, 2007; Jimenez-Lopez et al., 2020; Bilal et al., 2021). Hydroxytyrosol is one of the main polyphenols of EVOO. It has anti-inflammatory and antioxidant properties, reducing oxidative stress and the activation of inflammatory cells (Marcelino et al., 2019). Oleuropein is a glycosylated seco-iridoid, a phenolic bitter compound with antioxidant and anti-inflammatory activities (Ahamad et al., 2019).

Based on this background, the present study was conducted to evaluate the effect *in vitro* of EVOO, hydroxytyrosol, and oleuropein against two *H. pylori* strains and the effect *in vivo* of the oral administration of EVOO on the gastric mucosa of BALB/c mice infected with this microorganism. This work will allow us to understand the effectiveness of EVOO, and its main phenolic constituents, in eradicating *H. pylori* infection.

## Materials and methods

### Chemicals, reagents, and oil samples

All chemicals, unless stated otherwise, were purchased from Sigma-Aldrich Chemical Inc. (St. Louis, MO, United States). Hydroxytyrosol (HT) and Oleuropein (Olp) were supplied by Extrasynthèse (Lyon, France). These polyphenols were dissolved in a solution containing 6.7 mM Na_2_HPO_4_, 6.7 mM KH_2_PO_4_, 137 mM NaCl, 2.7 mM KCl, 0.8 mM CaCl_2_, 0.5 g/l albumin, and 1 g/l glucose, adjusted to pH 7.2, and stored at -20°C until required. The stock solutions were then diluted to the desired final concentration with the same solution. An extra virgin olive oil commercially available at the international level was selected for the study (EVOO) due to its high total antioxidant power. A sunflower oil (SFO) commercially available in Argentina was used for comparative studies due to significantly lower total antioxidant power compared to EVOO. The EVOO used in this work had the following composition: *Fatty acid profile* Myristic: 0.00% Myristoleic acid: 0.00% Palmitoleic: 13.91% Palmitoleic: 1.31% Margaric: 0.11% Heptadecenic: 0.23% Stearic: 2.05% Oleic: 69.59% Linoleic: 11.17% Arachidic: 0.38% Eicosaenoic: 0.38% Behenic: 0.11% Erucic: 0.00% Lignoseric: 0.00% Nervonic: 0.00% Pentadecanoic: 0.00% Arachidonic: 0.00% Eicosapentanoic: 0.00% Docosapentanic: 0.00% Docosahexaenoic: 0.00% *Sterols profile* Cholesterol: 0.15% Brassicasterol: <0.05% Cholesterol: 4.49% Campesterol: 4.49% Stigmasterol: 0.82% Beta-Sitosterol: 93.66% Delta 7 Stigmasterol: 0.11% *Phenolic compounds/secoiridoids profile* Oleocanthal: 123 mg/kg EVOO Oleacein: 216 mg/kg EVOO Oleuropein aglycone: 215 mg/kg EVOO Ligstroside aglycone: 30 mg/kg EVOO Oleokoronal: 113 mg/kg EVOO Oleomissional: 88 mg/kg EVOO S-(*E*)-elenolide: 1054 mg/kg EVOO Hydroxytyrosol: 5.95 mg/kg EVOO Tyrosol: 3.07 mg/kg EVOO Cinnamic acid: 0.90 mg/kg EVOO Pinoresinol: 13.3 mg/kg EVOO Apigenin: 2.53 mg/kg EVOO

### Total antioxidant activity of oil samples

The antioxidant activity of EVOO and SFO was determined spectrophotometrically as a measure of radical scavenging activity using 2,2-diphenyl-1-picrylhydrazyl free radical (DPPH) (La et al., 2021). A control sample containing a volume of solvent (methanol) equivalent to oil was used to measure the maximum DPPH absorbance. Aliquots of 0.1 ml of 100 μM solution of 5% DPPH in methanol were mixed with 0.1 ml of each sample. Samples in triplicate were mixed and incubated at room temperature in the dark for 30 min. The absorbance at 517 nm was recorded to determine the concentration of residual DPPH. The percentage of inhibition of the maximal absorbance was calculated according to the following equation:


$$\text{\%inhibition}=\left[\left(A_{\text{DPPH}}–A_{\text{OIL}}\right)/A_{\text{DPPH}}\right]\times \text{1}00$$

in which *A* is the absorbance of DPPH and oil, respectively. EC50 values correspond to the concentration of the sample, which scavenge 50% of DPPH free radicals.

### Strains and culture conditions

Strain NCTC 11638 (kindly provided by Dra. Teresa Alarcón Cavero, Microbiology Service of Hospital Universitario de la Princesa, Madrid, Spain) and strain HP661 (clinical strain, isolated from the gastric mucosa of a human patient) were used. Both *H. pylori* strains were grown in Mueller- Hinton agar (MHA) supplemented with 7% horse blood (MHA-HB) at 37°C under microaerophilic conditions and identified by microscopy, urease, catalase, and oxidase tests. A bacterial suspension of 1–1 × 10^8^ colony forming units for milliliter (CFU/ml) was prepared for *in vitro* assays.

### Animals and experiment protocol

Male adult BALB/c wild-type (WT) mice were used (*n* = 60). Three independent experiments were carried out with 5 mice *per* group (*n* = 20 mice *per* experiment). Animals were kept under a 12-h dark/light cycle in a temperature-controlled room (24–25°C) with free access to drinking water and laboratory food. All animal experiments were evaluated and approved by the Institutional Committee for Care and Use of Laboratory Animals (IACUC), Universidad Nacional de San Luis (Protocol No. B-328/19). Regulations of this Committee are in strict accordance with the recommendations in the Guide for the Care and Use of Laboratory Animals of the National Institute of Health (NIH, United States) to comply with established international regulations and guidelines.

### *In vitro* antimicrobial activity of oil samples, hydroxytyrosol, and oleuropein

**FIGURE 1:**
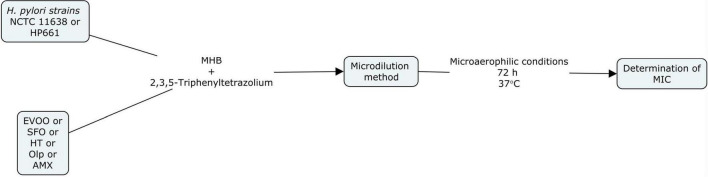
Flow diagram of the *in vitro* study of extra virgin olive oil (EVOO), sunflower oil (SFO), hydroxytyrosol (HT), oleuropein (Olp), and amoxicillin (AMX) against *Helicobacter pylori* strains NCTC 11638 and HP661 (Behzadi and Gajdács, 2021).

**FIGURE 2:**
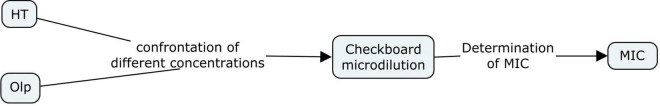
Flow diagram of the *in vitro* study of different concentrations of hydroxytyrosol (HT) together with oleuropein (Olp) against *Helicobacter pylori* strains NCTC 11638 and HP661 (Behzadi and Gajdács, 2021).

The anti-bacterial activity of EVOO, SFO, HT, and Olp against *H. pylori* strains was assayed by broth microdilution method using Mueller Hinton Broth (MHB) according to CLSI (2007) guidelines. Serial dilutions of amoxicillin (AMX) (Sigma-Aldrich Co., St Louis, MO, United States) were used to control the susceptibility test. Broth microdilution methods were carried out in 96-well microtiter plates. Aliquots (100 μl) of each extract dilution and each bacterial suspension adjusted to a scale of 0.5 in MacFarland’s standard [1 × 10^8^ colony forming units (CFU)/mL] were dispensed into each well. Two hundred microliters of extract, bacterial suspensions, MHB, and 0.9% saline were also included. Plates were incubated in microaerophilic conditions at 37°C for 3 days. The results were evaluated by colorimetric evaluation using 2,3,5-Triphenyltetrazolium chloride (TTC) as an indicator. Minimal inhibitory concentration (MIC) was measured by determining the smallest amount of extract or antibiotic needed to inhibit the visible growth of the microorganism. The Checkerboard microdilution test was applied to measure the probable synergistic effect of HT and Olp (Martinez-Irujo et al., 1996). The checkerboard assay was performed by mixing the two chemicals in different concentrations and determining the MIC for HT and Olp. A positive control with AMX was performed. All tests were performed in duplicate (Figures 1, 2).

### Experimental protocol and *Helicobacter pylori* infection for *in vivo* studies

**FIGURE 3:**
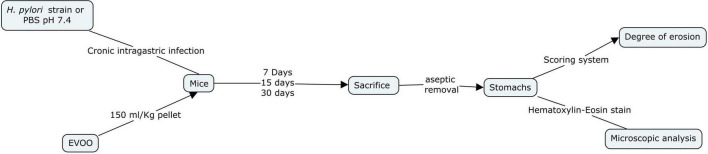
Flow diagram of the *in vivo* study of extra virgin olive oil (EVOO) against the infection of *Helicobacter pylori* strains NCTC 11638 in the gastric mucosa of mice (Behzadi and Gajdács, 2021).

Mice were distributed into four groups: (1) Control (C), (2) *H. pylori*-infected (HP), (3) EVOO (E), and (4) HP + EVOO (HPE). The control group received the ordinary diet (without EVOO) and a sterile phosphosaline buffer solution (PBS × pH 7.4) instead of the microorganisms. EVOO was administered with food (150 ml EVOO/kg pellet), being mixed well with the ordinary diet, to EVOO and HP + EVOO groups. Animals in the infected groups were administered intragastrically with 300 μl of the microorganism suspension (1–5 × 10^8^ CFU/ml) every 3 days for 3, 15, or 30 days (Robinson et al., 2017). Mice were sacrificed by cervical dislocation 3, 15, and 30 days after the first administration of saline solution or *Helicobacter pylori* suspensions. Their stomachs were removed aseptically, opened along the greater curvature, and washed gently with ice-cold saline solution. Immediately after the collection, the degree of erosion was assessed with a scoring system and the observation of histological preparations for light microscopy (Figure 3).

### Scoring system

The degree of erosion was assessed from a scoring system designed by Marazzi-Uberti and Turba (Ohta et al., 2005) as follows: 0, no erosions; 1, 1–3 small erosions (4 mm diameter or smaller); 2, more than 3 small erosions or one large erosion; 3, one large erosion and more than 3 small erosions; 4, 3–4 large erosions; 5, any very large erosion or ulcer perforation. The results were expressed in terms of an ulcer factor, which is the average severity of erosions *per* mouse for each group on a scale from 0 to 5. The sum of these values was divided by the number of animals. This procedure was performed using a Nikon binocular stereomicroscope (×40 magnification).

### Light microscopy

The samples for light microscopy were fixed for 24 h in a 10% formaldehyde solution (prepared with saline solution, pH = 7), dehydrated in graded ethanol and xylol, and embedded in paraffin. Serial sections (6 μm) were mounted on glass slides and deparaffinized. Sections were stained with hematoxylin–eosin in order to evaluate the general histoarchitecture and the degree of gastric lesions.

### Statistical analysis

Statistical analysis was performed using GraphPad Prism version 5.00 for Windows and GraphPad In Stat version 3.00 for Windows (GraphPad Software,^1^ San Diego, CA, United States). All data are expressed as the mean ± standard error of mean (S.E.M). A probability of *p* < 0.05 was considered statistically significant.

## Results

### *In vitro* evaluation of the antibacterial effect of extra virgin olive oil, sunflower oil, hydroxytyrosol, and oleuropein against *Helicobacter pylori*

The results of this analysis are summarized in Table 1. Briefly, the antimicrobial effect of EVOO against both strains showed a MIC of 229.5 μg/ml. Separately, HT and Olp showed no effect at concentration assayed range 25–4000 μg/ml. Interestingly, the combination of both compounds assayed by the checkerboard method showed an inhibitory effect at values of 19.27 μg/ml (HT) + 2.1 μg/ml (Olp) against NCTC strain, while the values were 154.16 μg/ml (HT) + 8.44 μg/ml (Olp) against 661 strain. On the other hand, no inhibitory effect was observed with SFO (Table 1). Total antioxidant power values of oil samples can be seen in Table 2.

**TABLE 1:** Minimal inhibitory concentration (MIC) of the extra virgin olive oil (EVOO) and the combined effect of hydroxytyrosol (HT) and oleuropein (Olp).

*H. pylori* strain	MIC EVOO (μ g/mL)	MIC H + O (μ g/mL)	MIC AMX (μ g/mL)
NCTC 11638	229.5	19.27 + 2.1	0.5
HP661	229.5	154.16 + 8.44	0.25

This table shows the concentration in μg/ml of EVOO and the combination of HT and Olp that had an inhibitory effect against *Helicobacter pylori* strains.

**TABLE 2:** The values of EC50 represent the average of total antioxidant power of each sample oil, with their standard deviation.

Oil sample	EC50	MIC AMX (μ g/mL)
EVOO	1.7 ± 0.01	0.5
SFO	3 ± 0.02	0.25

The higher the EC50, the lower the total antioxidant power. Results are expressed as mean ± SEM.

### Macroscopic evaluation after the *in vivo* action of chronic administration of extra virgin olive oil on the gastric mucosa in animals infected with *Helicobacter pylori*

**FIGURE 4:**
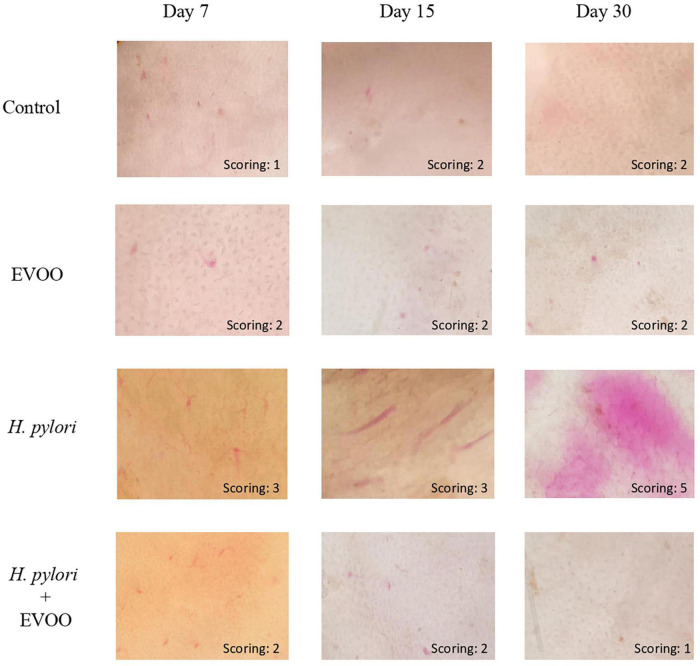
Macroscopic evaluation of the gastric mucosa surface of mice after 7, 15, or 30 days of treatment from four experimental groups: (1) Control, (2) Extra virgin olive oil (EVOO), (3) *Helicobacter pylori*, and (4) EVOO + *H. pylori*. Gastric mucosa from the control and EVOO groups shows a healthy appearance, with a score of 1 and 2 (control) and 2 (EVOO) at different infection times. *Helicobacter pylori* group had an increasing scoring with the time, reaching the highest value at 30 days, while in the group infected and treated with EVOO, the gastric mucosa structure is similar to that of the control and EVOO groups.

**FIGURE 5:**
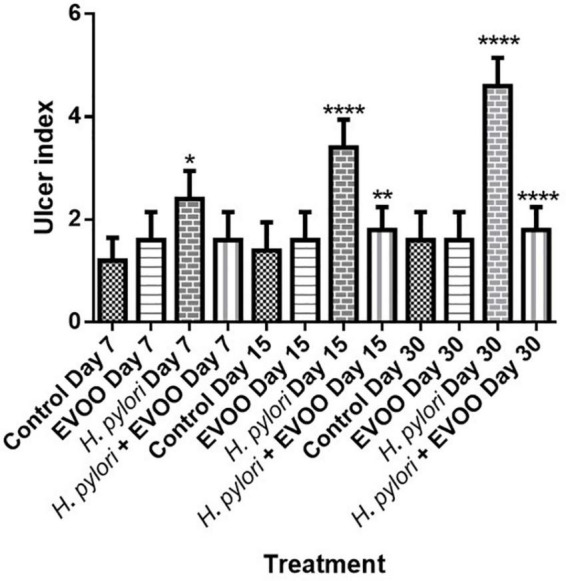
Effects of extra virgin olive oil (EVOO) on gastric lesions induced by the infection of *Helicobacter pylori* in mice. Asterisks denote significant differences between groups (*p < 0.05, **p < 0.01, and ****p < 0.001). All values are expressed as mean ± S.E.M.

Figure 4 shows representative images of the gastric mucosa surface after 7, 15, and 30 days of treatment from four experimental groups: (1) Control, (2) EVOO, (3) *H. pylori*, and (4) EVOO + *H. pylori*. The gastric mucosa surface from the control and EVOO groups shows a healthy macroscopic appearance with score values of the ulcerogenic index of 1 and 2 (control) and 2 (EVOO). The ulcerogenic index of the *H. pylori* group’s stomachs is significantly higher than that of the control and EVOO groups at 7 and 15 days (*p* < 0.005 and *p* < 0.0001) (Figure 5). Gastric mucosa from infected animals and without EVOO show the presence of small and medium-size erosions that were increasing, both in number and size, reaching the highest level (5) after 30 days from the first infection (*p* < 0.0001). Regions with mild, intense hyperemia are observed. In contrast, the group infected and treated with EVOO exhibited the presence of few erosions, which decreased in number as the treatment progressed. Hyperemia was not observed at any of the times analyzed in this group (*p* < 0.0001) (Figure 5).

### Histological analysis

**FIGURE 6:**
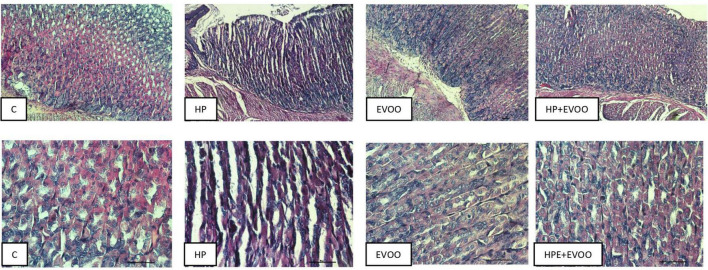
Hematoxylin–Eosin stain at 7 days after infection. The mucosa of the control and extra virgin olive oil (EVOO) groups shows normal histological characteristics. The mucosa of animals infected with *Helicobacter pylori* shows apparent injuries, mainly disruptions of the lining epithelium and damage in the isthmus and the neck of gastric glands. A decrease in the size of the gland epithelial cells can be observed. Blood, remnants of injured tissue, and some mucus filaments are observed in the lumen. The mucosa of animals infected with *H. pylori* and treated with EVOO shows morphological characteristics similar to those of normal and EVOO mucosa, even though slight damage is observed. Scale bar: 40 μm.

**FIGURE 7:**
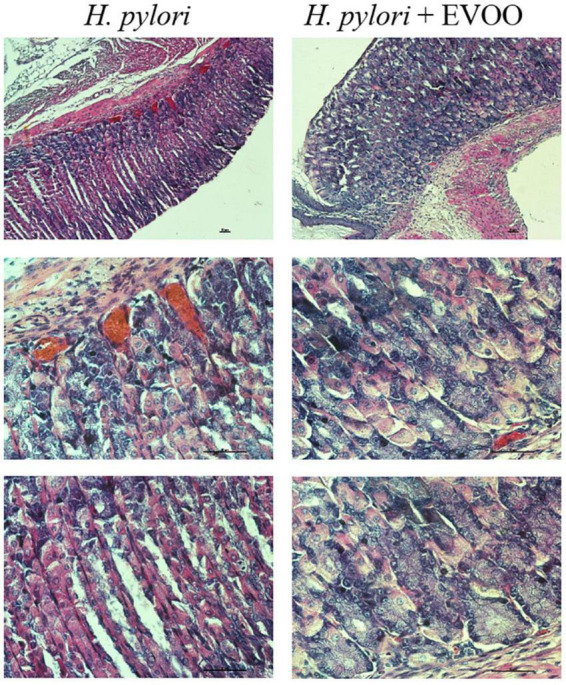
Hematoxylin–Eosin stain of *Helicobacter pylori* group and *H. pylori* and treatment group at 15 days. The mucosa of animals infected with *H. pylori* shows structural alterations, such as an increase of the gland gastric, vasodilation, and increased blood supply. The mucosa of animals infected with *H. pylori* and treated simultaneously with EVOO show morphological characteristics similar to those of normal and EVOO mucosa, even though slight vasodilation is observed. Scale bar: 40 μm.

**FIGURE 8:**
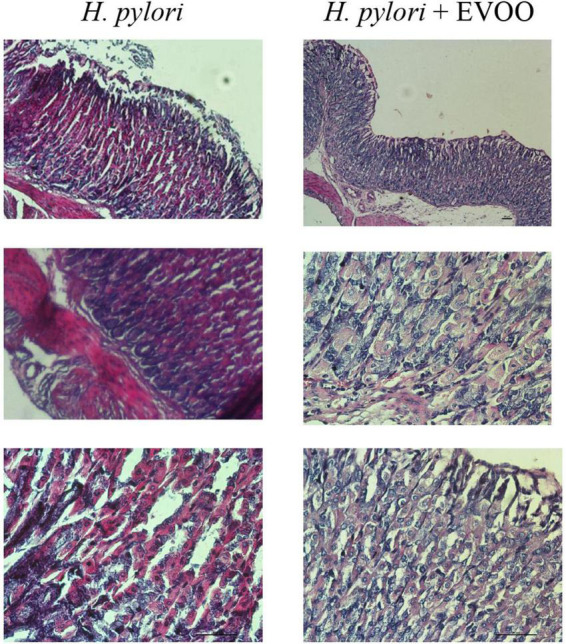
Hematoxylin–Eosin stain of *Helicobacter pylori* group and *H. pylori* and treatment group at 30 days. Significantly damaged tissue is observed with many blood vessels in gastric mucosa from the *H. pylori* group. At 30 days, significantly damaged tissue is observed with the presence of a large number of blood vessels. The mucosa of animals infected with *H. pylori* and treated with EVOO shows morphological characteristics similar to those of normal and EVOO mucosa. HP + EVOO group shows a cytoprotected gastric mucosa. Scale bar: 40 μm.

Figures 6–8 show micrographs, at different magnifications, of histological sections of hematoxylin–eosin-stained mouse stomachs on days 7, 15, and 30 after infection, respectively. In these images, it is possible to observe the histoarchitecture of the gastric mucosa representative of the different experimental groups. The mucosa of the control and EVOO groups shows normal histological characteristics at the three times studied. It is possible to observe the lining epithelium, the glandular epithelium, the lamina propria, and the *muscularis mucosae* without structural alterations. The mucosa of animals infected with *H. pylori* at 7 days shows apparent injuries, mainly disruptions of the lining epithelium and damage in the isthmus and the neck of gastric glands. A decrease in the size of the gland epithelial cells can be observed. Blood, remnants of injured tissue, and some mucus filaments are observed in the lumen. At 15 days post-infection, an increase in the stomach lumen with vasodilation and increased blood supply is observed. Infiltration of inflammatory cells can also be seen. At 30 days, significantly damaged tissue is observed with the presence of a large number of blood vessels. The mucosa of animals infected with *H. pylori* and treated with EVOO shows morphological characteristics similar to those of normal and EVOO mucosa, even though slight damage is observed at 7 days, which increases at 15 days. However, the cells are in better condition compared to the HP group.

## Discussion

*Helicobacter pylori* is a microorganism that produces chronic gastritis, peptic ulcers, and gastric neoplasms in the human stomach mucosa (Matongo and Nwodo, 2014; Huang et al., 2017; Park et al., 2018). Due the localization of *H. pylori*, drugs must penetrate the gastric mucosa layer and it is due to this, that in the treatments two to three antibiotics (metronidazole, amoxicillin, or tetracycline) are used, among a proton-pump inhibitor is used, but most of the time, the elimination is not successful due to the antibiotic resistance, patient compromise, and age (Castro et al., 2012; Ranjbar et al., 2017).

Today, natural products and derivates are being studied due to their different properties. Extra virgin olive oil is a vegetable oil obtained from olive fruit by a mechanical process without any solvents and at a temperature that does not degrade it. It contains monounsaturated fatty acids, primarily oleic acid, carotenoids, sterols, lycopene, and hydrophilic phenolics such as hydroxytyrosol and oleuropein (Osman et al., 2017; Negm et al., 2020). Different properties have been described for EVOO, like antioxidant, anti-inflammatory, anti-cancer, anti-atherogenic, hypoglucemic, hepatic-, cardiac-, and neuro-protective, anti-viral, and anti-bacterial (Barbaro et al., 2014).

This work aimed to study the effect of EVOO and its main phenolic compounds (hydroxytyrosol and oleuropein) *in vitro* against *H. pylori* strains and evaluate the action of the administration of EVOO *in vivo* using an animal model on the gastric mucosa infected with *H. pylori*.

Castro et al. (2012) studied the effect *in vitro* in three *H. pylori* strains of olive oil extracts in PBS. Starting from an initial inoculum of 10^6^ CFU/ml, where after 5 min of contact, all the strains were killed, and no growth was observed. These extracts contained high levels of dialdehyde form of decarboxmethyl elenolic acid linked to tyrosol and dialdehydic form of decarboxymethyl elenolic acid linked to hydroxytyrosol, in a concentration of 30–60 μg/ml, but also, the last wash show effect, where the concentration was 5–10 μg/ml. In our study, the initial concentration was 10^8^ UFC/ml, and the strains were put in contact with different concentrations of EVOO using the board technique, where no growth was observed at 229 μg/ml. The differences with respect to our results may be due to the fact that the extracts were rich in certain compounds, while in this study, pure extra virgin olive oil was used.

Romero et al. (2007) studied the *in vitro* effects of phenolic compounds of EVOO against *H. pylori*, where the extract that had nine polyphenols, including hydroxytyrosol and dialdehydic form of decarboxymethyl oleuropein aglycon (Hy-EDA), showed bactericidal effect time-dependent in three of the eight strains tested. But when isolated compounds were tested against the most resistant strain of *H. pylori*, none of them showed significant bactericidal effects except Hy-EDA. This accompanies our results, where no effect was observed on the part of the compounds separately, but synergy was seen when hydroxytyrosol and oleouropein were put in contact at the concentrations of H = 19.27 μg/ml + O = 2.1 μg/ml against NCTC strain, while in 661 strain, the values were H = 154.16 μg/ml + O = 8.44 μg/ml.

Castro et al. (2012) also studied the effect of the administration of washed olive oil for 14 days in patients, using the ^13^C-urea breath test to confirm the presence or absence of *H. pylori*. In two different trials, several patients were abandoned because of the taste or nausea. In both cases, only the 26 and 10% showed eradication of *H. pylori*, and several patients who had been negative for *H. pylori* were positive after one month of treatment. In our study, using an animal model of mice, the infection of *H. pylori* in the stomach mucosal was demonstrated by histology, and the group of mice who were infected and subsequently administered EVOO continuously not only showed eradication of the microorganism but also an improvement in the damage to the gastric mucosa, with a resolution of the ulcers.

Gastric ulcers are lesions in the gastric mucosa that extend along the *muscularis mucosae*, which are characterized by different stages of necrosis, neutrophil infiltration, reduced blood flow, increased oxidative stress, and inflammation (Siwe et al., 2021). Bhattamisra et al. (2018) had similar results in the treatment of ulcers with geraniol, with a reduction of ulcers in comparison with infected animals with *H. pyl*ori. Our results show a protective effect in the formation of ulcers in the first days of infection, having and keeping a scoring of 2 (*p* < 0.005), while the animal infected show an increase in the number and size of ulcers (*p* < 0.0001) (Figure 5).

Extra virgin olive oil is rich in polyphenols, an important group of polar components that have numerous biochemist activities like preventing and inhibiting radical reactions in the human body. Free radicals cause oxidative damage to biomolecules like lipids and DNA, increasing the risk of chronic diseases (Cicerale et al., 2012; Nazzaro et al., 2019), and it has been shown that the ingestion of food rich in polyphenols radically decreases the generation of hydroxyperoxides. It has been observed that reactive oxygen species (ROS) and lipid peroxidation are involved in the gastric ulceration (Palacios-Espinosa et al., 2014) and that *H. pylori* produce urease, an enzyme that hydrolyzes urea to ammonia that provides, and leads to pH elevation, favoring gastric colonization and providing protection from the stomach hydrochloric acid, creating an alkaline microenvironment favoring the Michael reaction, where some free radicals could interact with cysteine, lysine, and histidine within proteins which could have consequences in the cells of the stomach (Matongo and Nwodo, 2014; Cherkas and Zarkovic, 2018). This could explain the effect observed in this work of the administration *in vivo* of EVOO in mice infected with *H. pylori*, where the inhibition of ROS and lipid peroxidation or the stimulation of antioxidants reduce ulcer and help the healing process. Over 30 days, in the beginning, was observed a decrease in the size of the epithelial cells of the stomach, followed in the following days by vasodilation with increased blood supply, observing in the last days great damage to the tissue and the presence of ulcers, but in the group infected and have the administration of EVOO in the diet, show a decrease in damage over 30 days.

In addition to all this, inflammation is a defense of the organism that involves the local changes, with vasodilatation and migration of inflammatory cells. It is documented that the phenolic compounds of EVOO have anti-inflammatory effects. Osman et al. (2017) studied the anti-inflammatory effects of EVOO compared to ibuprofen in the treatment paw of male mice, and a decrease in the volume of the inflammation was seen compared with controls but was lower than the mixture of EVOO with ibuprofen. In our results, the administration of EVOO over time shows an improvement in the tissue compared to the infected group, where the epithelial cells are in better condition, and there is not as much tissue disruption.

In conclusion, the current study shows, for the first time, the effect *in vitro* and *in vivo* of extra virgin olive oil against *H. pylori* using an animal model. Hydroxytyrosol and oleuropien had no effect on their own against *H. pylori*, but they did show effects in a combination of both. At the same time, EVOO showed *in vitro* effect against both strains. Also, in mice chronically infected with *H. pylori*, the administration of EVOO protects the gastric mucosa avoiding the formation of small erosions and ulcers. Future studies are needed to establish the mechanism of EVOO’s action at the gastric mucosa level in order to propose this product as a natural antimicrobial agent for the treatment of gastric *H. pylori* infections.

## Data availability statement

The raw data supporting the conclusions of this article will be made available by the authors, without undue reservation.

## Ethics statement

The animal study was reviewed and approved by Institutional Committee for Care and Use of Laboratory Animals (IACUC), Universidad Nacional de San Luis (Protocol No. B-328/19).

## Author contributions

AA, MM, AV, and AP: study ideation, design, and manuscript–critical revision. AA, AV, and AP: sample collection, preparation, and manuscript–initial draft. AA: sample processing, experimentation, and statistical analysis. AP, MM, and AA: data acquisition and measurements. AV and AP: funding acquisition. All authors contributed to the article and approved the submitted version.

## Footnotes

1
www.graphpad.com
